# Sexual dimorphism as a facilitator of worker caste evolution in ants

**DOI:** 10.1002/ece3.9825

**Published:** 2023-02-15

**Authors:** Chris R. Smith

**Affiliations:** ^1^ Department of Biology Earlham College Richmond Indiana USA

**Keywords:** division of labor, drone, sexual selection, social insect

## Abstract

Ant societies are primarily composed of females, whereby labor is divided into reproductive, or queen, and non‐reproductive, or worker, castes. Workers and reproductive queens can differ greatly in behavior, longevity, physiology, and morphology, but queen–worker differences are usually modest relative to the differences in males. Males are short‐lived, typically do not provide the colony with labor, often look like a different species, and only occur seasonally. It is these differences that have historically led to their neglect in social insect research, but also why they may facilitate novel phenotypic variation – by increasing the phenotypic variability that is available for selection. In this study, worker variation in multivariate size–shape space paralleled male–queen variation. As worker variation increased within species, so did sexual variation. Across species in two independent genera, using head width as a proxy for body size, sexual size dimorphism correlated with worker polymorphism regardless of whether the ancestral condition was large or small worker/sexual dimorphism. Mounting molecular data support the hypothesis that queen–worker caste determination has co‐opted many genes/pathways from sex determination. The molecular evidence, coupled with the observations from this study, leads to the hypothesis that sexual selection and selection on colony‐level traits are non‐independent, and that sexual dimorphism may even have facilitated the evolution of the distinct worker caste.

## Graphical abstract

The male caste has been largely neglected in the study of ant morphological evolution. This manuscript shows evidence that variation in ant sexual dimorphism is associated with the degree of worker polymorphism. In order to understand female caste evolution, it is necessary to take a broad view of all castes and stop ignoring males.

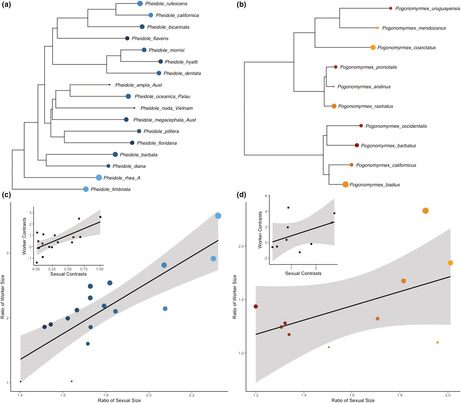

## 1 INTRODUCTION

**FIGURE 1:**
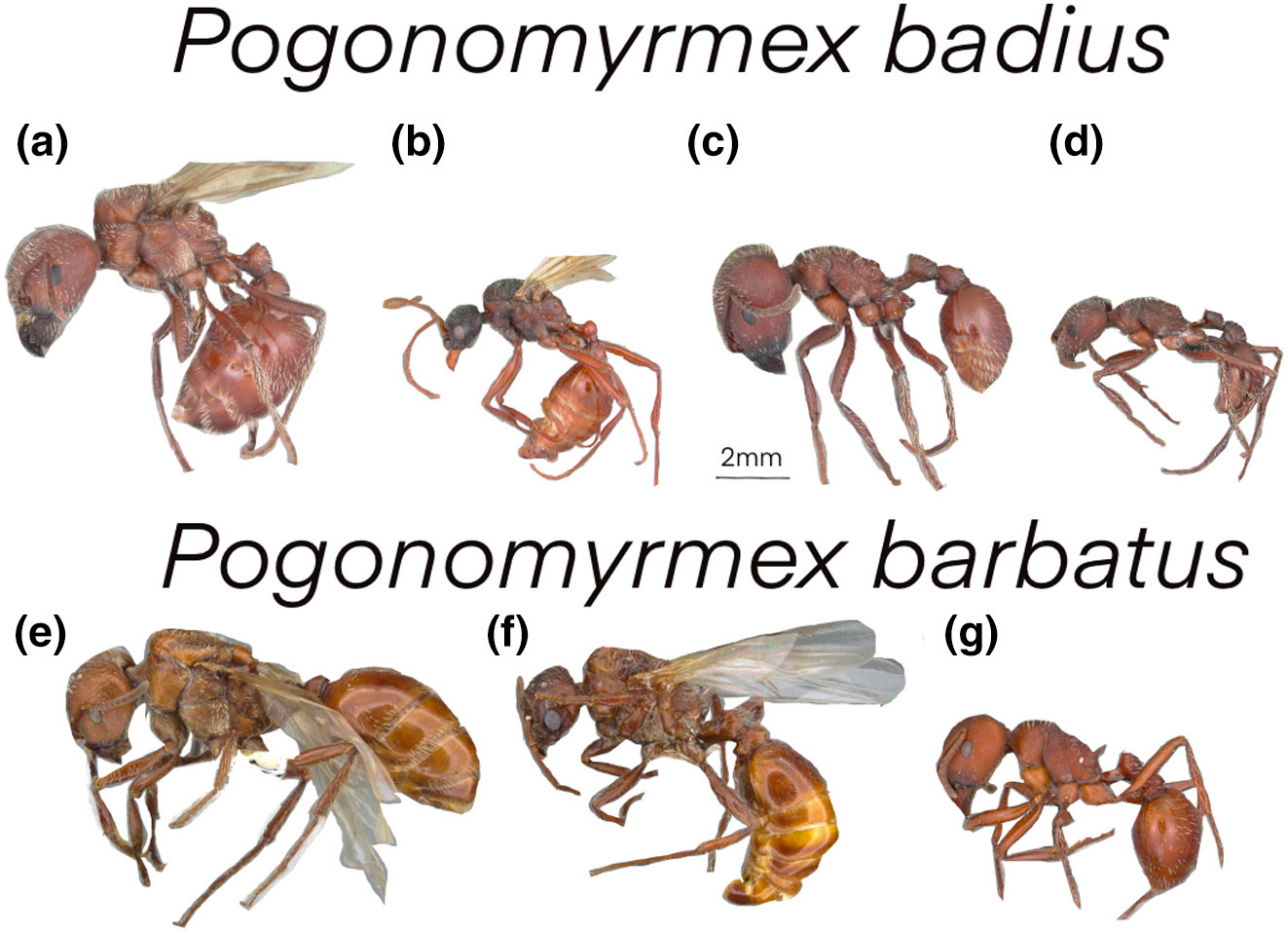
Images of *Pogonomyrmex badius* and *Pogonomyrmex barbatus* from www.antweb.org size scaled for comparison (note scales on images). a and e are gynes, b and f are males, and c, d, and g are workers. Images a–d were photographed by a. Nobile, e–f by m. Esposito, and g by j. Fogarty. In order of letters a–g, specimen codes are casent0104422, casent104421, casent0103057, casent0103056, casent0914095, casent0914094, and casent0102894.

Male ants are ephemeral in the annual cycle of a colony, and typically do not contribute work. They have been likened to “sperm missiles” (a statement attributed to E. O. Wilson) – a transient but essential function – delivering the male gametes of the superorganism to gynes so that the gynes can become queens and find new colonies, beginning the colonial life cycle anew (Hölldobler & Wilson, 1990). Because of this, males are often unseen and unsampled. Males are also haploid, typically developing from unfertilized eggs (males can be fathers but have no father). Males are thus genetically and behaviorally drastically different from the standard (female) ant, and their morphology is also clearly distinct as they typically have small heads with big eyes, a large muscly thorax with wings, and an abdomen tipped with an intromittent organ rather than a stinger. Males are clearly different than females; so different to look like a different species (Figure 1). And so, it is clear why males are largely ignored in the empirical and theoretical literature on caste and polyphenism in social insects – they are different.

While there are many papers and books written on social insect castes, most have no (or no meaningful) mention of males (e.g., Oster & Wilson, 1979). Because males and females share the vast majority of their genome (all in the case of the haplodiploid social insects), and because most genes are pleiotropic between the sexes (e.g., Artieri et al., 2009), the evolution of males and females is non‐independent. Thus, it stands to reason that male evolution is affected by, and affects, female evolution – including the evolution of the social insect castes.

Recent studies on gene expression during development have revealed that queen–worker caste determination shares many genes in common with male–female sex determination (Johnson & Jasper, 2016; Klein et al., 2016; McAfee et al., 2019; Roth et al., 2019; Warner et al., 2019). In fact, in both an ant and honeybee, there was a large suite of shared gene expression patterns between males and workers to the exclusion of gynes (Warner et al., 2019). Furthermore, in a CRISPR gene knockout of early sex determination genes in honeybee, it was shown that the *Fem* (feminizer) gene appears to control gonad size regardless of sex (although it also interacts with sex determination)(Roth et al., 2019). The emerging picture is that worker caste differentiation (from queens) arose via the co‐option of genes involved in sexual differentiation. That is, workers might be a gene expression developmental mosaic (Molet et al., 2012) of males and females.

The view that male‐ness contributed (and contributes) to worker evolution is logical. Male and female ants, despite behavioral, genetic, and morphological dissimilarities, arise from the same genome, whether haploid or diploid. Thus, the raw material for natural selection to work from is the combination of phenotypes expressed by both males and females; the colonial lifestyle of social insects may also buffer novel developmental mosaics from strong selection (Molet et al., 2012). Sexual dimorphism is present in most clades of Hymenoptera at least partly because of morphological and physiological specialization by females for parasitism/predation and brood care (in some lineages; Stubblefield & Seger, 1994). Due to sexual dimorphism, there was morphological variation for selection to act on – possibly enough to recombine developmental programs and produce the worker, a novel phenotype/caste that changed ecological and evolutionary history (Smith & Szathmary, 1997). In this study, I examine whether there is evidence for sexual dimorphism explaining variation in worker caste polymorphism, and address the following question: does the degree of sexual dimorphism correlate with variation in the worker caste? In other words, does male‐ness have a hand in the evolution and elaboration of the worker caste?

The relationship between sexual dimorphism and worker polymorphism was tested in two ways. First, detailed morphological measurements were taken in two related species that differ in both sexual dimorphism and worker polymorphism. Second, a broader phylogenetic approach was taken to examine the correlation between these variables across many species. The first approach examines the caste variation in morphological size–shape space and can be used to infer how castes differ over a developmental scale, while the second approach examines the same phenomenon at an evolutionary scale.

Two species of *Pogonomyrmex* harvester ant, *P. barbatus* and *P. badius*, were used to examine the morphological size–shape space across all adult castes. These species were chosen because sufficient samples were available across all castes for detailed measurement and because they differ in regard to both sexual and worker dimorphism. *P. barbatus* has very little sexual dimorphism while in *P. badius*, gynes are more than three times the size of males by dry mass and have an energetic cost to the colony of 4.5× of a male (Smith & Tschinkel, 2006). Similarly, workers of *P. barbatus* are characterized as monomorphic while those of *P. badius* are dimorphic, with clearly distinct major and minor worker sub‐castes (Tschinkel, 1998).

I hypothesized that the worker caste of *P. barbatus* would be similar in shape (i.e., changes in trait–space independent of size) to both males and gynes, as opposed to the traditional notion that females should clearly group to the exclusion of males. In *P. badius*, the gynes and major workers are very similar in basic visual appearance (shape and size) and appear to be co‐regulated at the colony level – that is, the production of gynes and major workers is similar as a function of colony size. While *P. badius* minors and males are not immediately clearly similar upon visual inspection, they also appear co‐regulated at the colony level (Smith, 2007; Smith & Tschinkel, 2006). And thus, for *P. badius*, it was hypothesized that increasing sexual dimorphism would be mirrored by increased worker dimorphism, with males/minors (small castes) and gynes/majors (large castes) grouping together in size–shape space.

In order to generalize the patterns seen across the two *Pogonomyrmex* species, where sexual dimorphism covaried with worker dimorphism, a generic‐level comparison was conducted across two genera that have broad variation in sexual and worker dimorphism, *Pogonomyrmex* sensu stricto (Taber, 1998) and *Pheidole*. These two genera were also chosen as they met two other important criteria, (1) a high‐quality molecular phylogeny was available, and (2) sufficient samples of all castes were available for measurement on Antweb.org. Based on the prior data on the two *Pogonomyrmex* species, it was hypothesized that there would be a positive correlation (controlling for evolutionary history) between sexual dimorphism and worker di/polymorphism. The use of these two genera has the potential to provide a strong hypothesis test for this correlation as the ancestral state in each case is opposite. The ancestral *Pogonomyrmex* species (sensu stricto) have monomorphic workers and little sexual dimorphism, while the ancestral state of *Pheidole* is to high worker and sexual dimorphism.

## 2 METHODS

### 2.1 *Pogonomyrmex* body measurements

*Pogonomyrmex barbatus* and *P. badius* were used for measurements as a large quantity of individuals were available from previous field and lab sampling. Samples used for measurement were either stored at −80°C (most *P. barbatus*) or dried (*P. badius*); for the former, individuals were from years of field and lab sampling, and some of the same colonies as were part of previous studies (Smith et al., 2012, 2015, 2018) and similarly for the latter (Smith, 2007; Smith & Tschinkel, 2006). For both species, the objective was to maximize variation in body size for each caste rather than maintain sufficient sampling to examine colony‐level effects. *P. barbatus* lab colonies produced microgynes and micromales as well as minim/nanitic‐like workers, sometimes after years of lab rearing. These micro‐individuals were included to help disentangle the effects of size and caste. Because size variation was the primary variable being maximized in the sampling design, some colonies are only represented by single individuals while others with many. A total of 31 colonies of *P. barbatus* were included spread across 25 gynes, 19 males, and 38 minor workers (82 individuals total). A total of 11 *P. badius* colonies were sampled with 17 gynes, 23 males, 21 major workers, and 23 minor workers (74 individuals total).

All ants measured were dried and separated into body and appendage segments using a stereo microscope, and glued to card stock (on their right side) to help standardize focal plane and angle among samples. All measurements were done using a stereomicroscope (SZX7; Olympus) with an attached camera (Retiga 2000R, Q‐Imagine) and measured after calibration using iSolutions‐Lite software (iMT Technology); magnification of images differed for body parts and castes but ranged from 25 to 56×.

**FIGURE 2:**
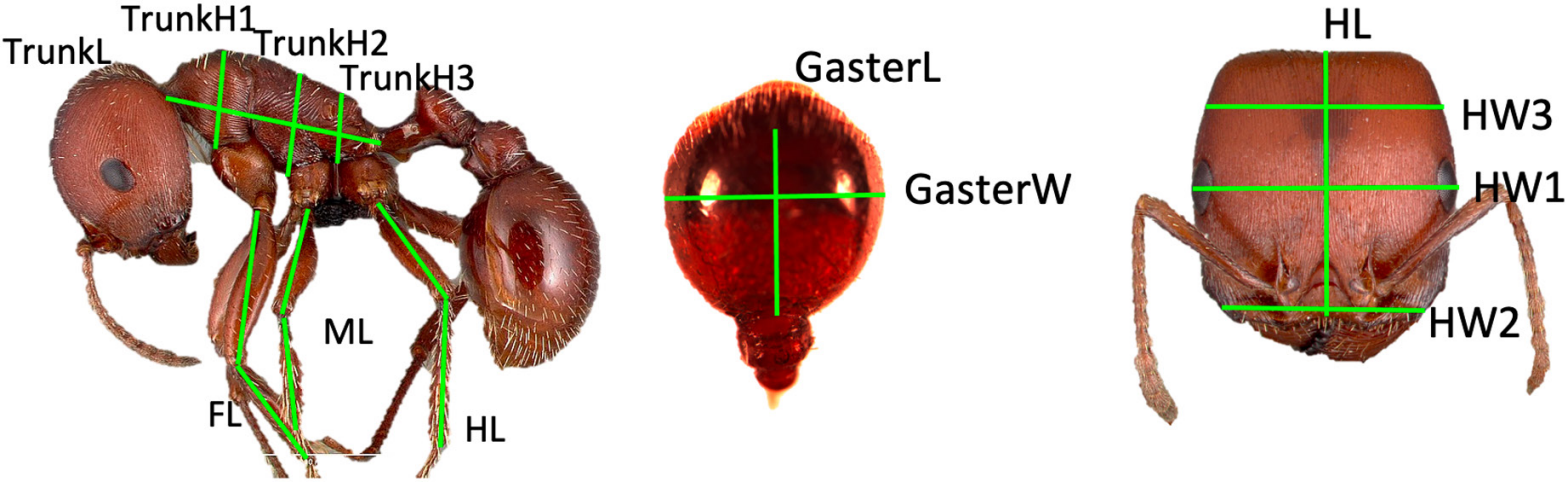
Measurements taken on each specimen. *Note:* However, that while the head and full profile images in this figure are from Antweb.org (*Pogonomyrmex barbatus* specimen casent0006306), measurements were done on individual body segments that were separated and glued to card stock. The left profile and right‐most head were imaged by M. Esposito; the middle image of the gaster was by M. Akhter.

Body parts measured in *Pogonomyrmex* were chosen for their repeatability and frequency of use in ant studies in order to facilitate cross‐study comparisons. Pictures and descriptions of these standard measures are available at https://www.antwiki.org/wiki/Morphological_Measurements (edited by S. Shattuck) and in Figure 2. A total of 16 measurements were taken for each ant. Body part abbreviations are as in Figure 2. (1) Head length (full‐face view, edge of clypeus to head vertex, HL); (2) mesosoma/Weber's length (profile view, head attachment to petiole attachment, TrunkL); (3) gaster length (dorsal view, post‐petiole attachment to the end of first gastral tergite along post‐petiole‐to‐sting axis, GasterL); (4–9) the length of the tibia and femur of each leg (fore, mid, and hind) was measured (FL, ML, HL); (10) head width 1 (full‐face view, head width across the eyes, HW1); (11) head width 2 (full‐face view, width at clypeus, HW2); (12) head width 3 (full‐face view, width at mid‐point between vertex of head and eyes, HW3); (13) gaster width (dorsal view, widest point of first gaster segment perpendicular to length axis, GasterW), and (14–16) thorax height at three points (profile view, measured at the anterior top of each leg, fore, mid, and hind, TrunkH1–H3). Ant length (sum of head, mesasoma, and gaster length) was used as a proxy of ant size when comparing to multivariate statistics (below).

All analyses were done using R 4.1.2 (R Core Team, 2021) and the RStudio interface (RStudio Team, 2022). Plots were constructed using ggplot2 (Wickham, 2016) and where relevant combined using ggpubr (Kassambara, 2020).

Non‐metric multidimensional scaling (NMDS) was used to compare body size–shape across the measured characters in both *P. barbatus* and *P. badius*. Bray–Curtis dissimilarities were used (Euclidian distances gave similar results) and only two dimensions were extracted for this analysis – the metaMDS function from the package vegan 2.5 (Oksanen et al., 2020) was used, and then points were extracted and plotted.

Relationships in multivariate space, among and within castes, were assessed using linear regression. Because body size is typically the largest predictor of variation in trait comparisons, it was expected that MDS1 would be correlated with body ‘size’. Body length (the sum of the length of the three body segments) was used as a proxy for body size and tested as a predictor of MDS1. MDS2 is orthogonal to MDS1 (“size”) and is sometimes referred to as “shape” because it is the factor with the greatest amount of variance explained independent of size; however, “size” and “shape” are concepts that do not have universal definitions. For the purpose of hypothesis testing, MDS1 will be evaluated as corresponding to “size” and MDS2 to “shape,” in the size–shape space of the larger hypothesis, although for accuracy I will refer to these as MDS1 and MDS2, respectively.

Differences among castes in multivariate (size–shape) space were assessed statistically using PERMANOVA using the function “adonis” from vegan (Oksanen et al., 2020) with pairwise comparisons made using adonis.pair from the EcolUtils package (Salazar, 2022). ANOVA was used to test for differences among castes in MDS2 (“shape”).

### 2.2 Measurements from Antweb.org

Measurements of worker and sexual head widths were done on specimens from Antweb.org; images were downloaded and measured using ImageJ2 v2.3.0 (Rueden et al., 2017) relative to the scale printed on the image. Head width was defined as the width of the head as measured across the eyes. A table of sample sizes of all images is available as a supplement (Table S1). The specimen identifiers are included with the measurement of each sample in the archived data (https://doi.org/10.5061/dryad.xksn02vks).

To ascertain whether the data available on Antweb.org were representative of natural variation, *Pogonomyrmex* harvester ant samples from Antweb.org were compared to head width measurements that were available from other sources. The overlap of worker size variation as measured from Antweb.org was compared to that reported in a taxonomic overview of the genus in North America (Cole, 1968). A *t*‐test was used to test whether the proportion of overlap between worker size ranges from Antweb.org and Cole (1968) differed from 1. Note that *P. wheeleri* measurements from Antweb.org (*N* = 2) did not overlap Cole's range and this 0% overlap was excluded from the analysis. To examine whether the amount of overlap of Cole's head width ranges with Antweb.org was affected by the number of samples available on Antweb.org, the proportion overlap per species was regressed against the sample size, per species, on Antweb.org.

Additional head width measurements were available from four species (*N* = 382 *P. badius*, *N* = 38 *P. barbatus*, *N* = 433 *P. coarctatus*, and *N* = 431 *P. rugosus*). These additional samples were measured optically using an SZX7 Stereomicroscope by Olympus with a Retiga 2000R camera by Q‐Imagine and iSolutions Lite software by iMT Technology; samples were from various colonies and populations sampled as parts of other studies by the author. Overlap of Antweb.org measurements with these more exhaustively sampled species was qualitatively compared.

### 2.3 Phylogenetic contrasts

Only species with at least one measure from each caste on Antweb.org were used in the analysis. See Table S1 for sample size information; identification information is available for each sample in the archived data (doi:10.5061/dryad.xksn02vks). Only species with sufficient sampling in Antweb.org (as above) and that were included in the available phylogeny (below) were included – exceptions and species substitutions are detailed below. The worker ratio was the largest divided by the smallest worker head width, while the sexual ratio was the mean gyne head width divided by the mean male head width. The gyne‐to‐worker ratio was calculated as the average gyne size divided by the minimum worker size.

Relationships of size variation between castes were tested using a linear model with phylogenetic correction. Phylogenetic correction was done using independent contrasts as calculated using the crunch function in the caper package in R (Orme et al., 2018). The genus‐level phylogenies used for phylogenetic correction were (Moreau, 2008) for *Pheidole* and (Johnson & Moreau, 2016) *Pogonomyrmex*. One sample of *Ph. megacephala* was excluded from analysis due to the likelihood that the printed scale was incorrect; the measured head width of that sample was more than 2.5× that of others of the same species and caste. For *Pheidole*, closely related species with measurements in Antweb.org were substituted for those on the phylogeny that was not (well) represented in Antweb.org. Below is a list of the substitutions made where the references support the close relationship or synonymy between the species pair. The first name is that from Antweb.org and the second is the name from the phylogeny (Moreau, 2008): *Ph. vigilans – Ph. ampla* (Brown Jr., 1971), *Ph. creightoni – Ph. californica* (Burge, 2005)*, Ph. williamsi – Ph. diana* (Wilson, 2003), *Ph. navigans – Ph. flavens* (Sarnat et al., 2015), *Ph. fervens – Ph. oceanica* (Wilson & Taylor, 1967), and *Ph. arnoldi – Ph. rufescens* (Bolton, 1995). Overall, 17 species of *Pheidole* were included in the analysis and 10 species of *Pogonomyrmex*.

## 3 RESULTS

### 3.1 Species comparisons

**FIGURE 3:**
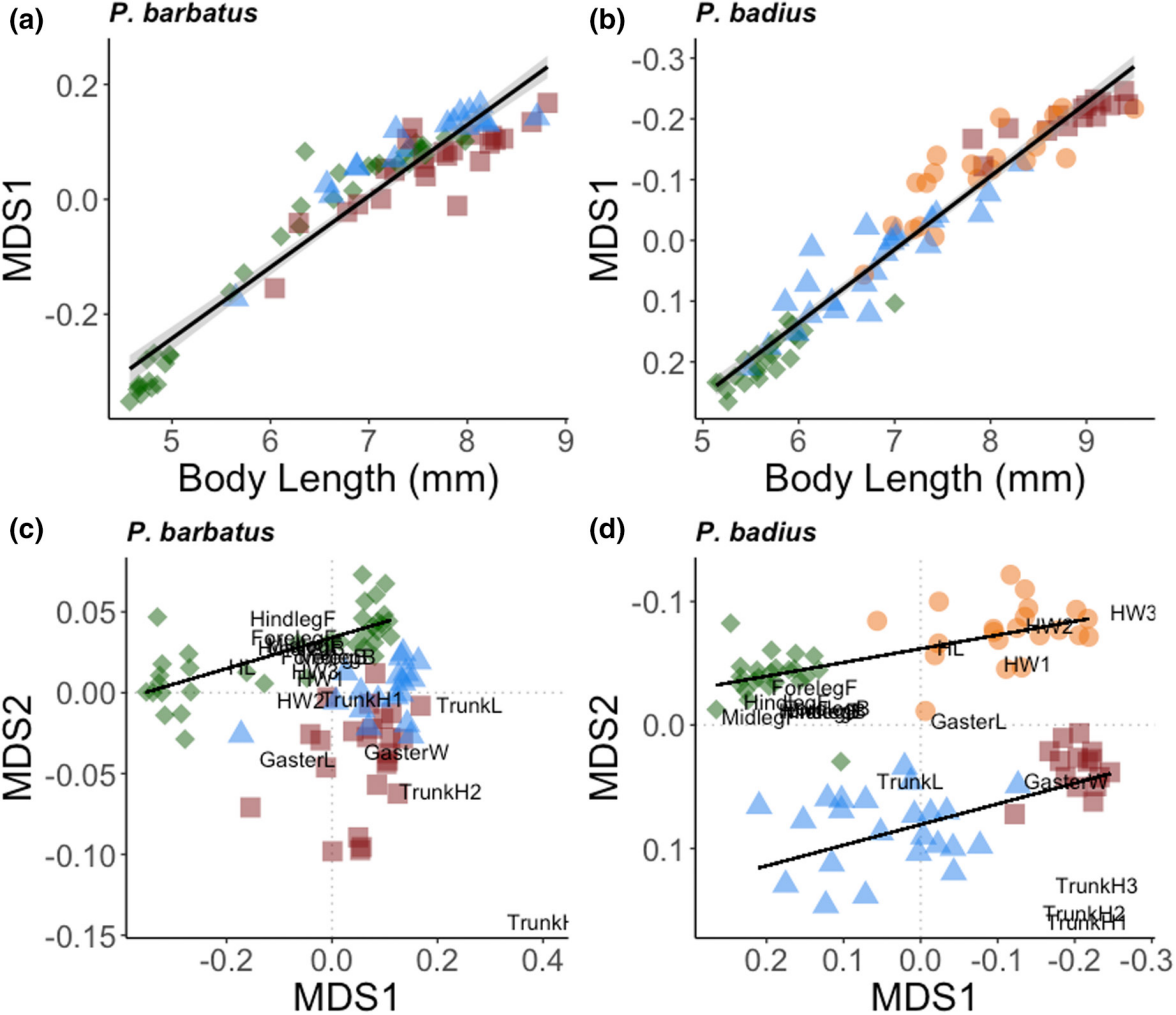
Body size by shape plots using nMDS for both *Pogonomyrmex barbatus* (left, a, c) and *Pogonomyrmex badius* (right, c, d). In figure (a, b), MDS1 is regressed onto untransformed body length to demonstrate that the first multivariate axis (MDS1) is a proxy for body size. Figure (c, d) thus show size on the *x*‐axis, and the first orthogonal multivariate factor (MDS2) is a dimension of shape. Superimposed on the plots is the correlation between individual measurements and the multivariate factors. In all plots, red squares = gynes, blue triangles = males, green diamonds = (minor) workers, and orange circles = major workers. The solid trend lines are statistically significant lines of best fit as reported in the text. *Note:* MDS1 was rotated in *P. badius* to make the plots easier to compare.

The first multivariate scaling axis is highly correlated with ant body length in both species, thus approximating size (*P. barbatus*: *F*_1,77_ = 757, *p* << 0.0001, *R* ^2^ = .91, *P. badius*: *F*_1,80_ = 1269, *p* << .0001, *R* ^2^ = .94) (Figure 3a,b). In both *P. barbatus* and *P. badius*, all castes were distinct in multivariate space (PERMANOVA: *P. barbatus*: *F*_2,76_ = 14.366, *p* < .001; *P. badius*: *F*_3,78_ = 89.881, *p* < .001; all castes were different from each other at *p* < .005 in both species in post hoc comparisons) (Figure 3c,d) as well as in MDS2‐“shape” (ANOVA: *P. barbatus*: *F*_2,76_ = 51.57, *p* << .0001; *P. badius*: *F*_3,78_ = 197, *p* << .0001; all castes were different from each other at *p* < .005 in both species in post hoc comparisons).

Qualitatively, as hypothesized, in *P. barbatus* with limited dimorphism, females (workers and gynes) did not group by MDS2 (“shape”) to the exclusion of workers, but rather males were intermediate between workers and gynes (MDS2 in Figure 3c). Contrary to the hypothesis, however, in the dimorphic *P. badius*, workers most differed from sexuals in MDS2 and each worker and sexual had distinct multivariate relationships (i.e., size–shape axes) (Figure 3d). Interestingly, the workers in *P. barbatus* do have a significant MDS1 by MDS2 relationship (“size” by “shape” relationship) (*F*_1,35_ = 44.52, *p* < .0001, *R* ^2^ = .55) while MDS2 is not predicted by MDS1 in males and gynes of *P. barbatus*. In *P. badius*, there is not a significant MDS1‐MDS2 (“size‐shape”) relationship in any caste, although visually this appears to possibly be a sample size artifact. That said, MDS1 (“size”) predicts MDS2 (“shape”) for each worker and sexual, and these slopes are parallel (total model: *F*_3,78_ = 183, *p* << .0001; size × shape: *p* < .0001; interaction: *p* = .14). Also very interestingly, the MDS1‐MDS2 (“size‐shape”) slope for *P. barbatus* workers is not different from that of *P. badius* workers, potentially suggesting conservation of the growth mechanisms governing shape change with size in this genus.

Predictable traits helped separate castes in each species; note, below, trait size as referenced is trait size accounting for body size. The distance from the origin in Figure 3c,d corresponds to the strength of correlation between each MDS axis and the measured characters. In both species, trunk/mesosomal height helped differentiate sexual castes due to the thoracic enlargement that accommodates wing muscle. Leg length (for all legs and leg segments) and head size tended to help separate workers from sexuals in *P. barbatus*, but helped separate minor workers/males from major workers/gynes in *P. badius*. Gaster size tended to help separate gynes in both species. These qualitative comparisons of castes by the measurements correspond to casual observation and common sense.

### 3.2 Antweb.org proof of concept

**FIGURE 4:**
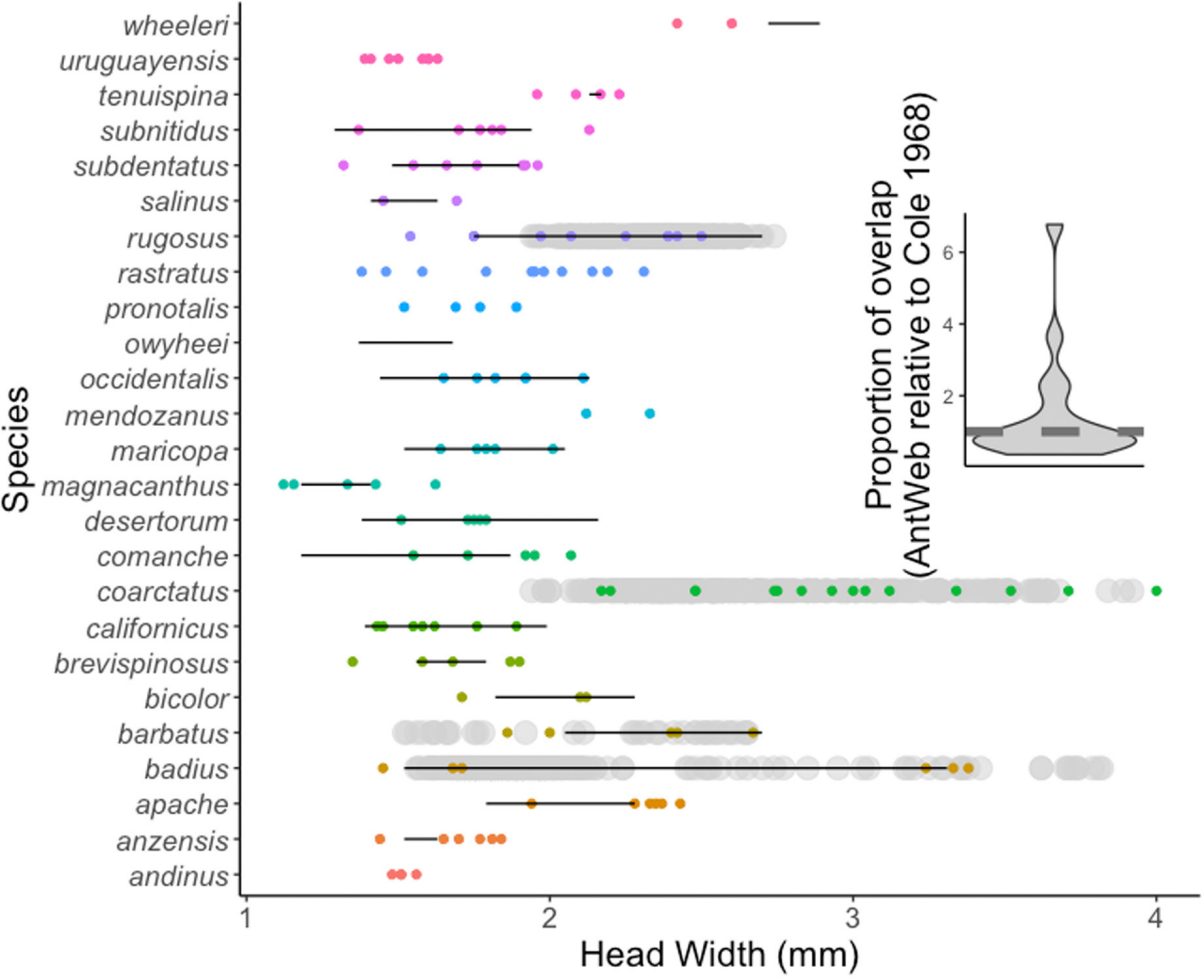
Head width measurements of workers from Antweb.org, the author, and the literature (Cole, 1984). Colored points are measurements from Antweb.org, background gray points are measurements by CR Smith, and lines represent the min and max head size as reported by Cole (1984). The inset is a violin plot showing the distribution of worker size range overlap as measured from Antweb.org and by Cole (1984). An overlap of 1 would mean that both sources report the exact same size range for a species; an overlap of less than one means that Antweb.org underestimates variation relative to Cole. The proportion overlap between these two sources is not different than 1 (*t* = 1.26, df = 16, *p* = .23). The measurements present on Antweb.org are largely representative of worker variation in the species examined.

Overall, the specimens available on Antweb.org seem to be relatively good estimates of the natural variation present in these species (Figure 4). The proportion of overlap between Antweb.org and the worker head width ranges reported by Cole (1984) does not differ from 1 (*t* = 1.26, df = 16, *p* = .23). The degree of overlap between Cole (1984) and measurements from Antweb.org is not affected by the number of samples measured (*F*_1,15_ = 1.65, *p* = .22). It should be noted, however, that several species on Antweb.org show a much greater degree of variation (200%–600%) compared to Cole (1984) (Figure 4 inset). When the Antweb.org sample range is compared to four intensively measured species, there is 70%–88% overlap; Antweb.org measurements show more variation in one of the four species (*P. rugosus*, Figure 4).

### 3.3 Generic comparisons

**FIGURE 5:**
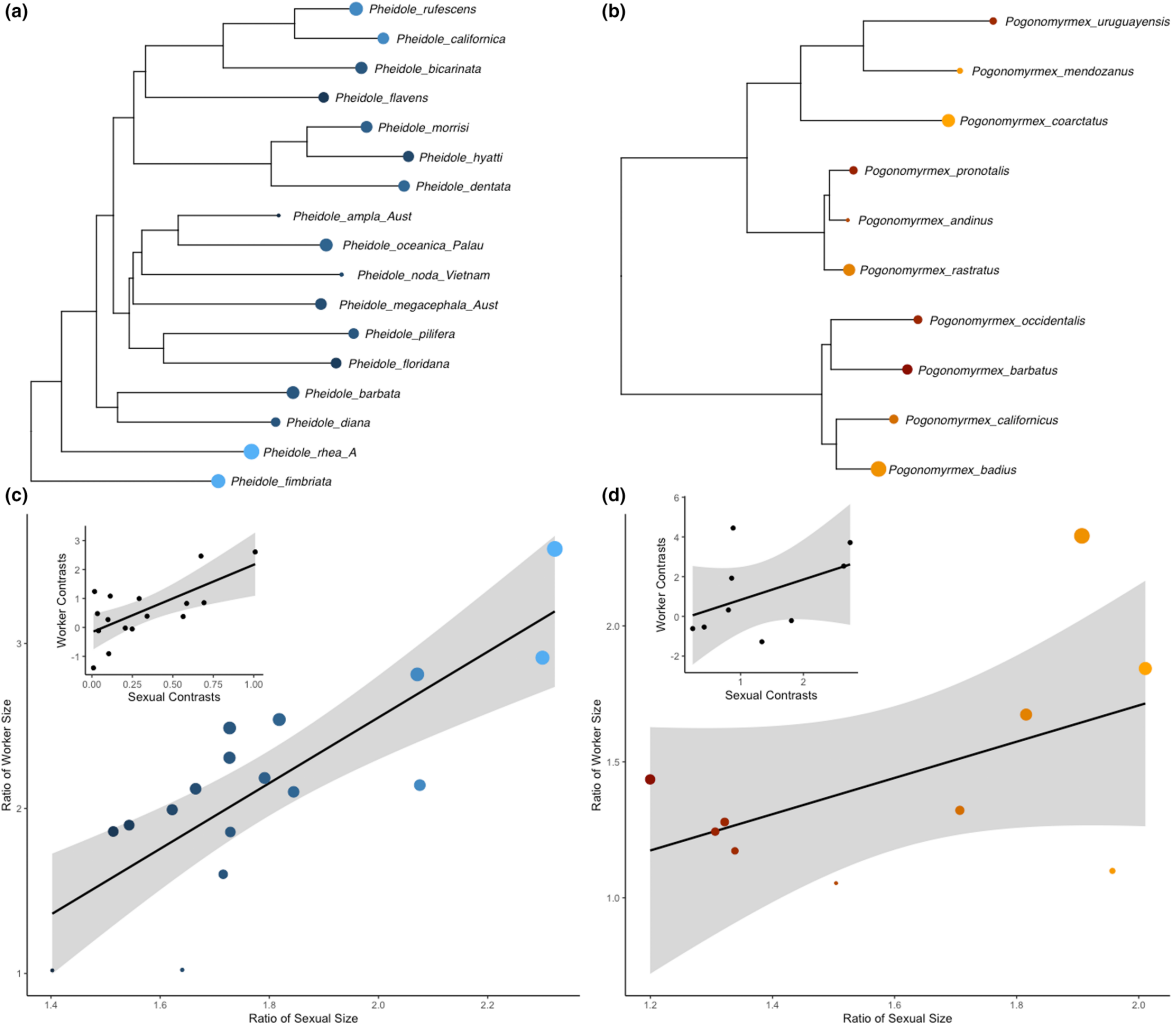
Phylogenies of *Pheidole* (a) and *Pogonomyrmex* (b), along with scatterplots (c, d) showing the relationship between sexual size ratio (gyne/male size) and worker size ratio (largest/smallest size). Graph insets are plots of phylogenetically independent contrasts between sexual size ratio and worker size ratio. Point size is proportionate to worker ratio and color is proportionate to sexual size ratio (lighter color is larger). Phylogeny topologies from figure (a) Moreau (2008) and (b) Johnson and Moreau (2016).

In both *Pogonomyrmex* and *Pheidole*, the sexual size ratio is a significant predictor of the worker size ratio, once controlling for phylogeny (*Pogonomyrmex*: *F*_1,8_ = 5.421, *p* = .048, *R* ^2^ = .33, *Pheidole*: *F*_1,15_ = 20.77, *p* = .0004, *R* ^2^ = .55) (Figure 5 inset). The gyne‐to‐worker size ratio is also a significant predictor of the worker size ratio in both genera – to a greater extent than the sexual size ratio (*Pogonomyrmex*: *F*_1,8_ = 9.283, *p* = .016, *R* ^2^ = .48; *Pheidole*: *F*_1,15_ = 70.23, *p* < .0001, *R* ^2^ = .81). To further understand what drives the observed patterns, male and gyne head width were assessed as predictors of worker size variation. Gyne head width in *Pheidole* was a significant predictor of worker variation but not in *Pogonomyrmex* (*Pogonomyrmex*: *F*_1,8_ = 2.29, *p* = .17, *R* ^2^ = .12, *Pheidole*: *F*_1,15_ = 8.70, *p* = .01, *R* ^2^ = .32); male head width, alone, was not a predictor of worker variation in either genus (*Pogonomyrmex*: *F*_1,8_ = 0.04, *p* = .85, *R* ^2^ = 0; *Pheidole*: *F*_1,15_ = 1.65, *p* = .22, *R* ^2^ = .04).

## 4 DISCUSSION

As hypothesized, sexual dimorphism covaries with worker polymorphism in the sampled taxa. This pattern is evident when comparing the size–shape space of individual species as well as at the level of entire genera. While quite logical that male and female characters do not evolve independently, even in the absence of sexual selection, males have largely been absent from empirical and theoretical studies of the hymenopteran social insect castes.

While this study documents how sexual dimorphism relates to worker polymorphism in two genera of highly social and very derived ants, it is logical to think that sexual dimorphism was important for the origin of the worker caste and all of its subsequent elaboration/modification. Because pronounced sexual dimorphism is likely the ancestral state of the Hymenoptera (Stubblefield & Seger, 1994), the ancestral size–shape space in the wasp ancestor of the ants was the phenotype space available for selection that ultimately resulted in the worker caste. After all, the differences among castes are primarily the difference in the timing, location, and dosage of a shared set of genes, and so the worker was/is, genetically, both part male and part female. For example, the data from Warner et al. (2019) show that there are exceedingly few genes that are uniquely expressed in any caste – <1% of genes have caste‐unique expression and all of those are rare in copy number (these data included transcriptomes of workers, males, and queens across developmental stages). Smith et al. (2015), similarly, found that most genes changed the direction of bias (between queen and worker) across development. These studies suggest that, at the level of genetic variation, ant castes are largely shaped by regulatory evolution. The genetic architecture to make a worker can exist even in the absence of worker production (Smith et al., 2015).

Variation in mating and social systems among ant species varies the degree of sexual selection and thus sexual dimorphism. Thus, it is hypothesized that there is a correlation between mating systems and worker polymorphism. It may even be that the observed correlation between worker caste variation and genetic diversity within nests (Anderson et al., 2008; Fjerdingstad & Crozier, 2006; Hughes & Boomsma, 2008; Smith, Anderson, et al., 2008; Smith, Toth, et al., 2008) that results from mating systems like polyandry is mechanistically confounded by the phenotypic results of sexual selection (e.g., sexual, or natural, selection on male body size to increase mating probability).

The causal arrow in the relationship between sexual and worker dimorphism likely points in both directions. As above, sexual dimorphism may have provided the raw material (size–shape) for the evolution of the worker caste, but selection on worker characteristics and the population of workers in colonies (colony‐level selection) (Oster & Wilson, 1979) likely facilitates/inhibits the phenotypic variation available to sexual selection. Strong natural selection on worker size variation, for example, increases the size–shape space and may facilitate the production of novel sexual forms. The head width of either sexual caste, alone, did not predict worker size variation, or at least when it did (queen head width in *Pheidole*); it was a weaker predictor than either the sexual size ratio or queen‐to‐worker ratio. This result highlights the likely importance of standing phenotypic variation (developmental potential) to affect size variation in the worker caste. That is, variation in size of one phenotype begets variation in size of an alternative phenotype.

The causal direction of the relationship is far less important than recognizing the non‐independence of the two processes. Selection on worker polymorphism, for example, increases the phenotypic space available to sexual selection, not necessarily whether that selection occurs. Selection for increased variation in the size–shape of any caste increases the raw material for selection – it increases opportunity. As previously theorized, the colony environment buffers its members from individual‐level selection and can be an incubator of sorts for novelty, especially via modifications of the social environment (Molet et al., 2012; Rajakumar et al., 2012).

Not surprisingly, queen–worker dimorphism was a better predictor of worker polymorphism than sexual dimorphism because female castes are developmentally more similar than the sexes. Previous studies have documented the correlation between queen–worker and worker polymorphism across species (Fjerdingstad & Crozier, 2006), although these factors are not always correlated (Lecocq de Pletincx et al., 2021). The development of workers and queens is more similar than that of workers and males (Anderson et al., 2008). For example, queen–worker differentiation takes place in early development while worker caste differentiation occurs later; note, the most upstream mechanisms of female caste determination can occur prior to fertilization or in early embryogenesis (Schwander et al., 2008), but most differentiation (Lillico‐Ouachour & Abouheif, 2017) and canalization (Qiu et al., 2022) occur later in larval development. Hymenopteran males and females differ genetically where male‐destined eggs are typically unfertilized, and at least in honeybees, the mechanism involves heterozygosity at a single locus, CSD, that then feeds into the conserved sex differentiation pathway of insects (Beye et al., 2003; Roth et al., 2019). Many studies are now demonstrating that sexual determination and differentiation and caste determination and differentiation are not mechanistically independent, both pathways utilize many of the same genes (Johnson & Jasper, 2016; Klein et al., 2016; Roth et al., 2019; Warner et al., 2019).

One of the most surprising results of this study is the relationship between MDS1 and MDS2 (morphometric size–shape space) in both *Pogonomyrmex* species. In *P. barbatus,* there is a “size‐shape” relationship among workers, and in *P. badius*, the slope for workers is parallel to that of sexuals and is similar (non‐differentiable) in slope to the worker relationship in *P. barbatus*. The major worker caste in *P. badius* is an evolutionary novelty in the sense that it is a unique and derived character for the species, completely absent in all other extant North American *Pogonomyrmex*. Based on the measurements taken, the major worker is simply an extension of the minor worker in this “size‐shape” space. The major caste, in other words, is simply a size increase of the minor worker using the same allometric growth rules – and the gyne is, in a sense, a size extension of the male using the same allometric growth rules – and surprisingly, workers and sexuals vary on parallel axes only differing in their starting size. This result suggests that, based on the linear measurements taken, all castes differ only slightly in basic developmental scaling. It should be noted, however, that developmental scaling is not likely the whole story as there can be discrete anatomical differences between castes (Boudinot et al., 2021) and more complex differences that are not captured by these rather simplistic measurements.

Clearly, including more genera and more body measurements is a logical next step to further evaluate the relationship between sexual dimorphism and worker polymorphism. Looking at more ant subfamilies will likely also reveal how generalizable this pattern is and how other aspects of biology constrain or facilitate caste evolution. Fortunately, the growing database of Antweb.org is an amazing resource and it seems that the maintainers of the database have done a commendable job of representing species‐level variation. Given the high quality and taxonomic breadth of the database, it is sure that it will be useful for the evaluation of additional macroevolutionary patterns in ants. In addition to databases of external morphology, there is now increased availability of micro‐CT‐scanned images of ants. The availability of three‐dimensional data for ant morphology and anatomy promises to add significantly to our knowledge of how castes differ in complex elements of internal anatomy and shape.

## AUTHOR CONTRIBUTION

C.R. Smith conceived the study, supervised all data collection, did all analysis, and wrote the manuscript.

## FUNDING INFORMATION

Funding for this project was provided by the Earlham College Summer Collaborative Research program via an Anonymous donor, the Gerald Bakker Collaborative Research Endowment Fund (at Earlham College), and the Department of Education's Ronald E. McNair Postbaccalaureate Achievement Program.

## Supporting information


Table S1

## Data Availability

All data and analysis code will be made publicly available upon manuscript acceptance at Dryad (https://doi.org/10.5061/dryad.xksn02vks).
